# Parental knowledge and practice on childhood caries prevention in northern Vietnam

**DOI:** 10.3389/fpubh.2023.1254479

**Published:** 2023-10-11

**Authors:** Dung Anh Vu, Hai Minh Vu, Hoang Minh Vu, Phuc Thai Tran, Hoang Huy Duong, Kham Quoc Tran, Bach Xuan Nguyen, Hien Xuan Luong

**Affiliations:** ^1^Department of Odonto Stomatology, Thai Binh University of Medicine and Pharmacy, Thái Bình, Vietnam; ^2^Department of Trauma, Thai Binh University of Medicine and Pharmacy, Thái Bình, Vietnam; ^3^Nursing Department, Thai Binh University of Medicine and Pharmacy, Thái Bình, Vietnam; ^4^Department of Neurology, Thai Binh University of Medicine and Pharmacy, Thái Bình, Vietnam; ^5^Faculty of Public Health, Thai Binh University of Medicine and Pharmacy, Thái Bình, Vietnam; ^6^VNU University of Medicine and Pharmacy, Vietnam National University, Hanoi, Vietnam

**Keywords:** knowledge, practice, dental caries prevention, children, parent

## Abstract

This study was conducted to describe the knowledge and practices on dental caries prevention among parents of preschool children in Vietnam and identify associated factors. A cross-sectional study was conducted in three preschools in Northern Vietnam in 2020. A total of 316 parents of preschool children were randomly recruited. Knowledge and practices regarding early dental caries prevention were asked by using a structured questionnaire. Multivariate Tobit regression was used to examine factors associated with knowledge and practice scores. Results showed four aspects of knowledge that had the lowest proportion of parents having correct responses included timing of complete primary tooth replacement (12.3%), benefits of undergoing regular dental examination (31.7%), technique for brushing a child’s teeth (33.9%), and duration for brushing (36.7%). The knowledge of parents was moderately low at 6.3/12 (SD = 2.3). The practices of parents were moderately good with the mean practice score at 6.1/9 (SD = 2.0). The proportion of parents taking children for regular checkups (56.2%) and replacing toothbrush every 3 months (53.7%) were the lowest. Information source, occupation, education and perceived necessity of oral care were found to be associated with parents’ knowledge and practices. To conclude, parents had moderate levels of knowledge and practices regarding early dental caries prevention in preschool children. Further studies and interventions should be performed to improve parental knowledge and practices that could enhance the oral health of children.

## 1. Introduction

Dental caries is highly prevalent among children worldwide (1). If not appropriately addressed, this condition possesses the capacity to progress to the dental pulp, resulting in discomfort for the affected individual and giving rise to localized complications associated with infection. Consequently, such complications have the potential to exert an impact on premature tooth loss, and impaired masticatory function, as well as overall physiological wellness such as weight reduction, respiratory issues, joint ailments, cardiovascular disorders, and sinusitis. Moreover, situations arise where the deciduous dentition is affected by decay and requires untimely removal, resulting in potential complications in the subsequent eruption or alignment of the permanent dentition (2).

Various studies have provided evidence regarding the varying incidence rates of dental caries among children aged 1 to 5 years on a global level, ranging from 22 to 69% (3–5). Sergio et al. in their meta-analysis showed that the prevalence of early dental caries was 48%, with the highest rate in the Oceania continent (82%), followed by Asia (52%), Americas (48%), Europe (43%) and Africa (30%) (6). Another study’s findings indicate that, within the demographic of children aged less than 36 months, the highest mean prevalence of the condition was observed in North America (31. 7%) and South Asia (30%). In contrast, the lowest mean prevalence was reported in sub-Saharan Africa (14. 3%). With children within the age range of 36 to 71 months, the East Asia/Pacific region exhibited the highest mean prevalence at 68.7%, followed closely by the Middle East/North Africa region with a prevalence of 66.2% (7).

The preschool stage marks the period when children exhibit the presence of mature primary dentition. During childhood, young individuals are capable of embarking upon the initial stages of teeth brushing, visual image identification, as well as guiding oral hygiene. Nonetheless, the prevention and management of oral diseases encounter numerous challenges as a result of the early stage of development. Furthermore, the efficacy of oral disease prevention during this stage is contingent upon the parental knowledge and practices on the instruction and supervision of children on their oral hygiene routines, including brushing and flossing, as well as ensuring regular dental check-ups (8, 9). Some studies showed that children whose parents have correct oral hygiene awareness and behaviors have a lower rate of tooth decay than children whose parents do not have proper oral care behaviors (10–12). Hence, comprehending the knowledge and practices of parents and caregivers assumes a significant role in the advancement of intervention programs intended to alter behavior and foster health promotion for enhancing the oral health of children. However, in literature, several studies have revealed a lack of parental knowledge on oral care (13, 14); meanwhile, others indicated substantial knowledge and exhibited positive attitudes, yet demonstrated suboptimal implementation of practices (15, 16). Nevertheless, the majority of studies have reached the consensus that educational programs and initiatives are necessary to enhance parents’ knowledge and understanding of oral health matters (13–16).

In Vietnam, dental caries is a substantial phenomenon. Prior reports in different settings showed that the prevalence of dental caries among children aged 2–5 years was 82.9 to 89.1% (17, 18). Despite the significance of dental problems in preschool children, research related to knowledge, attitude and practice in dental care among their parents is limited. A previous study showed that the percentage of parents with appropriate knowledge, attitudes and practices was 54.6, 63.0 and 42.3%, respectively (19). More updated evidence should be provided in other settings for developing further intervention plans, which might support improving the dental health of preschool children. Therefore, this study was conducted to describe the knowledge and practices on dental caries prevention among parents of preschool children in Vietnam and identify associated factors.

## 2. Materials and methods

### 2.1. Study settings and sampling procedure

In 2020, a cross-sectional study was conducted within a Northern Vietnamese province. After considering the demographic characteristics of the province and the number of children in each preschool, we decided to perform the study in three preschools, one in an urban commune and two in rural communes to have an approximately equal number of participants between both settings. The schools were randomly selected from the list that was provided by the authorities of the communes. Then, in each school, we invited parents whose children studied in the selected schools. The inclusion criteria encompassed parents who had children between the ages of three and 5 years old currently enrolled at the corresponding school, as well as obtaining their informed consent to partake in the study. The study entailed the participation of a total of 316 parents. The response rate achieved a substantial value of 96%.

### 2.2. Data collection method

A structured questionnaire was utilized as a data collection instrument during participant interviews. The investigators included members of the research team and medical students with rigorous training. Every data collector attended a two-day training course. The initial day of training encompassed the instruction of data collectors on research objectives, research subjects, the structure of questionnaires, as well as appropriate interviewing techniques. On the subsequent day, the investigators actively engaged in the pilot study. The researchers conducted a trial interview with a cohort of 10 parents who fulfilled the specified eligibility criteria and were distinct from the parents of children selected for inclusion in the study. This study enabled the participants to become acquainted with the procedures of conducting interviews and to gather data reliably.

The questionnaire was developed based on reviewing the current literature on the same topics, as well as being consulted by experts in the field of community oral care and oral care for children. First, we sought and reviewed items that were proposed in the prior studies and developed a list of different questions regarding knowledge and practices on dental caries prevention for preschool children (20), especially in developing countries (13, 16, 21, 22). To ensure face validity, we performed expert panels that provided support to remove unnecessary or duplicated items and shortened the list of items. Then, by piloting the questionnaire and receiving feedback from participants, we revised the questionnaire regarding cultural background, text, language and logical order of items. The questionnaire consists of the following information:

*Demographic characteristics*: gender of the respondent, the gender of their children, their year of birth, level of education, occupation, living location number of children as well as the education and occupation of their spouse.

*Oral health of children*: We collected data on information sources for oral care and history of children’s tooth decay. Moreover, the participants were instructed to assess the necessity of oral care for preschool children, with 0 = not necessary, 1 = necessary and 2 = very necessary.

*Knowledge of dental caries prevention*: This study used 12 questions to evaluate the knowledge of parents on dental caries prevention. They focused on the awareness of parents regarding the timing of permanent teeth eruption, dietary restrictions necessary to prevent dental diseases, habits that should be limited to minimize dental illnesses, appropriate age of tooth brushing initiation, recommended duration of tooth brushing, optimal brushing techniques, duration of brushing, factors contributing to tooth decay, and recommended preventive measures to safeguard dental health. Each accurately responded item was awarded a score of one point. The range of the total knowledge score encompassed values between 0 and 12 points. The attainment of higher scores corresponded to a greater level of knowledge. The Cronbach’s alpha of these items was 0.651, suggesting acceptable internal consistency.

*Practices on dental caries prevention*: Nine items about different dental caries prevention practices were asked. We evaluated the frequency with which parents remind their children about oral hygiene, including the number of times they brush their children’s teeth in a day, the frequency with which they remind their children to wash their mouths and brush their teeth after meals, and the frequency at which they take their children for regular dental check-ups. Moreover, we explore the type of toothbrush and cup used by parents, as well as the selection of toothbrushes for children and guidelines on when to replace these brushes and introduce toothpaste for children. Parents’ actions after being informed of their child’s tooth decay were also asked. The overall evaluation of practice performance was measured on a scale ranging from 0 to 9 points, with a higher score indicating better practices. The Cronbach’s alpha of these items was 0.667, suggesting acceptable internal consistency.

### 2.3. Statistical analysis

Collected data was reviewed, cleaned and entered on Epidata 3.1 software. Data were analyzed using STATA 14.0 software. Descriptive statistical methods were used to describe frequencies and percentages, the mean, and standard deviation. A multivariable Tobit regression model was applied to evaluate the factors related to knowledge and practice scores. A backward stepwise selection strategy was used. Variables with a value of p of the log-likelihood of less than 0.2 were included in the model. A *p*-value with a value <0.05 was used to consider the level of statistical significance.

## 3. Results

Table 1 shows that, among 316 parents, most of them were male (56.7%) with a mean age of 31.9 years old (SD = 5.2). The majority of them had above high school education (46.2%) and were officials (47.2%), lived in rural areas (51.6%). The major information sources for dental care included social networks (44.4%), Internet (41.6%) and medical staff (42.2%). The majority of participants perceived that oral hygiene for children was necessary/very necessary (97.8%).

**Table 1:** Demographic characteristics of parents.

Characteristics		Freq. (*n*)	Percent (%)
Respondent’s gender	Male	179	56.7
	Female	137	43.3
Gender of child	Male	185	58.5
	Female	131	41.7
Education	Middle school	59	18.7
	High school	111	35.1
	> High School	146	46.2
Spouse’s education	Middle school	51	16.1
	High school	119	37.7
	> High School	146	46.2
Occupation	Farmer/worker	52	16.5
	Officials	149	47.2
	Different	115	36.4
Spouse’s occupation	Free	115	36.4
	Workers/farmers	117	37.0
	Officials	41	13.0
	Different	43	13.6
Living location	Rural	163	51.6
	Urban	153	48.4
Information sources	Family	112	35.7
	Friends and relatives	86	27.3
	Colleague	68	21.6
	Television, newspapers	127	40.3
	Internet in general	131	41.6
	Social networks	140	44.4
	Medical staff	133	42.2
	Others	13	4.1
Children with previous tooth decay	Yes	158	50.2
	No	157	49.8
Necessity of oral hygiene for children	Not necessary	7	2.2
	Necessary	107	34.0
	Very necessary	201	63.8
Characteristics		**Mean**	**SD**
Age of respondents (years old)		31.9	5.2
Number of children		2.1	0.6

Regarding knowledge Table 2 shows the parent’s knowledge about dental caries prevention for children, Table 2 shows that tooth brushing age (81.3%), cause of tooth decay (71.8%), time of permanent teeth eruption (68.7%) and number of primary teeth (68.0%) were the most accurate knowledge. The mean knowledge score was 6.3/12 (SD = 2.3).

**Table 2:** Parent’s knowledge about dental caries prevention for children.

Correct content	Freq. (*n*)	Percent (%)
The time of complete replacement of primary teeth (6 to 13 years old)	39	12.3
The time of full primary teeth eruption (3–4 years old)	133	42.1
The number of primary teeth (20 teeth)	215	68.0
The time of permanent teeth eruption (6 years old and above)	217	68.7
Food needs to be limited to prevent dental diseases (different types of sweet food)	186	58.9
Bad habits to avoid to prevent dental disease (snack food, sweet food, biting pen, sucking rice, sucking fingers, breathing through the mouth, leaning on the chin, biting hard objects)	180	57.0
The age when tooth brushing starts (3 years old)	256	81.3
The manner to brush a child’s teeth (brushing in longitudinal axis, circular brushing, brushing all three sides of the teeth)	107	33.9
The best brushing time (after meals)	116	36.7
The results of regular dental check-ups for children (prevention of oral diseases, early detection and treatment)	100	31.7
The causes of tooth decay (not having good oral hygiene, eating snacks, eating candy, drinking soft drinks, etc., due to bacteria)	227	71.8
Measures to protect a child’s teeth (limit eating candy, limit drinking soft drinks, limit eating snacks, good oral hygiene, brush teeth with fluoride, have regular dental check-ups)	207	65.5
	Mean	SD
Knowledge score (0–12)	6.3	2.3

According to practice, Table 3 shows that the mean practice score was 6.1/9 (SD = 2.0). Overall, the practices of parents were good. The proportion of parents taking children for regular checkups (56.2%) and replacing toothbrush every 3 months (53.7%) were the lowest.

**Table 3:** Parents’ practices in dental care for children.

Correct content	Freq. (*n*)	Percent (%)
Take the child for treatment if he or she has tooth decay	257	81.6
Regularly remind the child about oral hygiene	252	80.0
Brush the child’s teeth 1–2 times a day	275	87.3
Regularly/always remind the child to rinse their mouth and brush their teeth after eating	201	63.8
Take the child for regular dental checkups 1–2 times a year	177	56.2
Use separate toothbrushes and toothcups between parent and child	199	63.2
Use a child’s toothbrush to clean the child’s teeth	292	92.7
Replace toothbrush every 3 months	169	53.7
Use toothpaste for children	256	81.3
	Mean	SD
Practice score (0–9)	6.1	2.0

Table 4 depicts factors associated with knowledge and practice scores. Regarding knowledge, participants who were officials had a lower score than those who were farmers/workers (Coef. = − 1.17; 95%CI = -1.83; −0.50). Participants who preferred friends/relatives and the internet as information sources had a higher knowledge score than those not. Meanwhile, for the practice score, a higher spouse’s education level and family as an information source were associated with a higher practice score. Participants whose spouses were officials had lower practice scores than those who were self-employed. Notably, parents having higher perceived necessity of oral hygiene for children had significantly higher knowledge (Coef. = 0.80, 95%CI = 0.45–1.14) and practice scores (Coef. = 0.89, 95%CI = 0.58–1.20).

**Table 4:** Factors related to knowledge and practice of parents in early dental caries prevention.

Characteristics	Knowledge score		Practice score	
	Coef.	95%CI	Coef.	95%CI
Child’s gender (Female vs. Male)			−0.29	(−0.68; 0.10)
Respondent’s gender (Female vs. Male)	−0.34	(−0.77; 0.09)		
Education level (vs. <High School)				
High School	0.12	(−0.50; 0.75)		
> High School	0.60*	(−0.03; 1.24)		
Spouse’s education level (vs. <high school)				
High School			0.03	(−0.55; 0.62)
> High School			0.64**	(0.05; 1.23)
Occupation (vs. Farmer/worker)				
Officials	−1.17***	(−1.83; −0.50)		
Other	−0.69*	(−1.41; 0.03)		
Spouse occupation (vs. Self-employed)				
Farmer/worker			−0.16	(−0.62; 0.30)
Officials			−0.79**	(−1.47; −0.12)
Other			−0.99***	(−1.59; −0.38)
Sources of Information (Yes vs. No)				
Family	−0.37	(−0.84; 0.09)	0.64***	(0.22; 1.07)
Friends, relatives	0.70***	(0.19; 1.22)		
Colleague			−0.49*	(−0.99; 0.01)
TV, radio	0.35	(−0.11; 0.81)		
Internet in general	0.64***	(0.17; 1.11)		
Social network	0.32	(−0.13; 0.77)		
Medical staff			0.84***	(0.44; 1.23)
Necessity of oral hygiene for children	0.80***	(0.45; 1.14)	0.89***	(0.58; 1.20)
Knowledge score			0.13***	(0.04; 0.21)
Practice score	0.20***	(0.07; 0.32)		

***p < 0.01, **p < 0.05, *p < 0.1.

## 4. Discussion

According to the World Health Organization, a comprehensive strategy to prevent tooth decay in children cannot be carried out independently but must involve the contributions of many stakeholders such as healthcare professionals, educators, healthcare providers; caregivers and the child (23). This study informed that Vietnamese parents had moderate knowledge and practices in early caries prevention regardless of living location. Moreover, several social determinants of knowledge and practice such as occupation, information sources and the perceived necessity of oral care would be useful for further interventions in the community to improve children’s oral health.

Results of this study show that parents had a moderate-low level of knowledge, which was similar to other studies in the world as well as in Vietnam when they showed that parents’ knowledge about dental care was limited (19, 24, 25). One prior study shows that the percentage of parents with good knowledge of several fundamentals of dental care was less than 50% (24). Notably, four aspects of knowledge that had the lowest proportion of parents having correct responses included timing of complete primary tooth replacement (12.3%), benefits of undergoing regular dental examination (31.7%), technique for brushing a child’s teeth (33.9%), and duration for brushing (36.7%). These rates were comparable to other studies in both developed and developing countries. In a study conducted in Australia, a mere 40% of parents recognized the inadequacy of daily brushing as a significant risk factor for the onset of tooth decay at an early stage (22). Another research conducted on parents in Malaysia demonstrated that only 50% of these adults possessed knowledge regarding the potential risk of tooth decay in preschool-age children who are below 2 years old (26). In a study conducted by a cohort of Qatari parents, it was observed that a significant proportion (64%) of maternal participants displayed a lack of knowledge regarding the appropriate timing of the initial dental appointment for their children (27). Another study among Saudi parents uncovered that a majority of 53% expressed a lack of inclination toward availing dental care services for their children during the initial year of their lives (28). This also indicated a significant lack of basic knowledge in oral health care for children in Vietnam. One possible explanation for this phenomenon can be attributed to the lack of dental health education programs for children in Vietnam. In practice, alongside oral health care programs in schools, there is an infrequent observation of comprehensive health education programs addressing this issue. One additional issue that we observed during this study is the lack of importance given by parents to dental care for preschool-aged children. This is due to the belief that primary teeth would be replaced by permanent teeth, and thus there would be no need to pay attention to the care of primary teeth. As a result, despite being fundamental knowledge in the care of children’s oral health, parents have yet to fully realize the significance of this issue. This is asserted through the correlation between awareness of oral care necessity and the level of knowledge in parents, that individuals with higher perceived necessity levels tend to possess higher levels of knowledge. Thus, it is evident that parents would benefit from the implementation of educational programs that increase their awareness of the importance of preschool children’s oral care, as well as provide assistance and guidance regarding the significance of fundamental issues such as timing of tooth replacement, or technique/duration for brushing teeth.

The findings of this study indicate that parents exhibited a moderately high level of practice, which is consistent with parental practices observed in various regions (21, 27, 29, 30). A significant proportion of parents provided accurate responses showed promising findings that further interventions should focus on informing accurate knowledge for them in order to provide fundamental for their accurate practices. However, several aspects of practices should be concerned such as regular checkups (56.2%) and replacing toothbrush every 3 months (53.7%). Indeed, regular health check-ups and toothbrush replacement play an important role in ensuring children’s oral health, maintaining oral hygiene and preventing the growth of bacteria inside the oral cavity, which are the main cause of dental caries and thereby tooth decay. Therefore, parents need to form these habits to help ensure children’s oral health. Results of the regression analysis indicate that parents who primarily rely on information from friends/relatives, the Internet, and medical staff regarding child dental care tend to possess a greater level of knowledge and practices compared to individuals who do not utilize these sources of information. The aforementioned information channels demonstrate potential avenues for subsequent intervention programs aimed at enhancing parents’ knowledge pertaining to children’s oral care.

Collectively, it is notable that the comprehension and implementation of dental care among parents is lacking, not only within Vietnam but also globally. Research findings indicate that a notable impact on children’s oral health is observed due to inadequate knowledge and awareness of dental treatment, oral hygiene, and dietary habits (30). There is robust evidence attesting to the effectiveness of assisting parents and caregivers in enhancing their knowledge, attitudes, and practices to advance children’s oral health (30–33). Henceforth, health education intervention programs must be implemented moving forward to enhance parental knowledge, attitudes, and practices on dental care for children.

The research possesses certain constraints. This study was solely undertaken within a single province in northern Vietnam, thus potentially limiting its applicability to current knowledge, attitudes, and practices of parents in different localities. The investigation employed a cross-sectional study design, which constrained the potential to establish a causal relationship. Finally, the items regarding knowledge and practices in this study were not fully validated. Further studies should be warranted to examine the validity of these questions as well as provide more evidence about the knowledge and practices of parents in oral care for their children, which is important for planning effective interventions to improve children’s health and well-being.

### Conclusion

Parents had moderate knowledge and practices regarding early dental caries prevention in preschool children. Further studies and interventions should be performed to improve parental knowledge and practices that could enhance the oral health of children.

## Data availability statement

The raw data supporting the conclusions of this article will be made available by the authors, without undue reservation.

## Ethics statement

The studies involving humans were approved by Thai Binh University of Medicine and Pharmacy. The studies were conducted in accordance with the local legislation and institutional requirements. The participants provided their written informed consent to participate in this study.

## Author contributions

DV: Conceptualization, Formal Analysis, Writing – original draft, Writing – review & editing. HaV: Conceptualization, Formal Analysis, Writing – original draft, Writing – review & editing. HoV: Conceptualization, Formal Analysis, Writing – original draft, Writing – review & editing. PT: Investigation, Writing – original draft, Writing – review & editing. HD: Investigation, Writing – original draft, Writing – review & editing. KT: Supervision, Writing – original draft, Writing – review & editing. BN: Formal Analysis, Writing – original draft, Writing – review & editing. HL: Supervision, Writing – original draft, Writing – review & editing.
